# Hands-on training in structural biology, a tool for sustainable development in Africa series 4

**DOI:** 10.1242/bio.059487

**Published:** 2022-08-16

**Authors:** Dinkorma T. Ouologuem, Fatoumata O. Maiga, Antoine Dara, Abdoulaye Djimdé, Daouda A. K. Traore, Emmanuel Nji

**Affiliations:** 1 Malaria Research and Training Center, Department of Epidemiology of Parasitic Diseases, Faculty of Pharmacy, University of Science, Techniques, and Technologies of Bamako, Bamako BP 1805, Mali; 2 BioStruct-Africa, 14343 Vårby, Stockholm, Sweden; 3 Faculty of Natural Sciences, School of Life Sciences, Keele University, Staffordshire ST5 5BG, UK; 4 Life Sciences Group, Institute Laue Langevin, Grenoble 38000, France; 5 Faculté des Sciences et Techniques, University of Science, Techniques, and Technologies of Bamako, Bamako BP E423, Mali; 6 Infection Program, Biomedicine Discovery Institute, Departments of Biochemistry and Molecular Biology, Monash University, Clayton 3800, Victoria, Australia; 7 Centre for Research in Therapeutic Sciences (CREATES), Strathmore University, Madaraka Estate, Ole Sangale Road, 59857-00200, Nairobi, Kenya

**Keywords:** BioStruct-Africa, Structural biology, Capacity building, Africa

## Abstract

Structural biology is an essential tool for understanding the molecular basis of diseases, which can guide the rational design of new drugs, vaccines, and the optimisation of existing medicines. However, most African countries do not conduct structural biology research due to limited resources, lack of trained persons, and an exodus of skilled scientists. The most urgent requirement is to build on the emerging centres in Africa – some well-established, others growing. This can be achieved through workshops that improve networking, grow skills, and develop mechanisms for access to light source beamlines for defining X-ray structures across the continent. These would encourage the growth of structural biology, which is central to understanding biological functions and developing new antimicrobials and other drugs. In this light, a hands-on training workshop in structural biology series 4 was organised by BioStruct-Africa and the Malaria Research and Training Center (MRTC) in Bamako, Mali, to help bridge this gap. The workshop was hosted by MRTC from the 25th to 28th of April 2022. Through a series of lectures and practicals, the workshop enlightened the participants on how structural biology can be utilised to find solutions to the prevalent diseases in Africa. The short training gave them an overview of target selection, protein production and purification, structural determination techniques, and analysis in combination with high-throughput, structure-guided, fragment-based drug design.

## Abstract (precis)

**Summary:** BioStruct-Africa has been building capacity in structural biology for Africa-based biologists and researchers.

## Introduction

Visualizing biological molecules at the molecular level is essential in understanding how they perform their function (Coincon et al., 2016; Nji et al., 2019a; Thulasingam et al., 2021). A structural biology approach captures the structure of macromolecules, such as proteins and nucleic acids, which could help elucidate their function and role in diseases. Obtaining such atomic-level structures and understanding how they perform their function could help design new drugs and vaccines and optimise them to provide better treatment options. However, apart from South Africa and, most recently, Kenya (a Wellcome Trust International Intermediate Fellowship: 222999/Z/21/Z to Dr Emmanuel Nji), no other African country conducts research in structural biology to the best of our knowledge. Therefore, it is clear that Africa lags behind in the field of structural biology, and the urge to fill this gap led to the establishment of BioStruct-Africa in 2017, which has been building capacity for Africa-based researchers in structural biology (Nji et al., 2019b).

BioStruct-Africa is a non-profit organization registered in Stockholm, Sweden (Swedish corporate ID: 802509-6689) whose mission is to build capacity for Africa-based researchers in the indispensable field of structural biology. The core team members are made up of, but not limited to, Africans with expertise in structural biology. BioStruct-Africa's inaugural workshop in 2019 (Nji et al., 2019b) was a satellite meeting coinciding with the second Pan African Conference on Crystallography (PCCr2) and the third African Light Source Conference (AfLS3). The workshop was structured to contain both introductory lectures and hands-on training in structural biology. BioStruct-Africa planned two workshops in 2021 in Rwanda and Malawi, but due to the COVID-19 pandemic, the workshops were unfortunately cancelled.

The current BioStruct-Africa workshop was held at the Malaria Research and Training Center (MRTC) from the 25th to 28th of April 2022. The MRTC within the University of Science, Techniques and Technologies of Bamako is divided into several research groups and/or units, including the Genomics and Molecular Epidemiology, B-cell Laboratory, Cellular Immunology Laboratory, Molecular Epidemiology and Drug Resistance Unit, Clinical Drugs, and Vaccines Development units, Data Management and Analysis Group, Diagnostic Laboratories and Entomology Groups. During the past 30 years, MRTC in collaboration with the US National Institutes of Health and various partners, has built state-of-the-art facilities for malaria and other pathogens research, including extensive −80°C freezer and liquid nitrogen storage facilities, parasite culture facilities, insectaries, genomic data storage and Bioinformatics facilities, etc.

The BioStruct-Africa workshop hosted by MRTC was a hybrid event consisting of both lectures (on-site and virtually) and hands-on training. The workshop's main goal was to give the participants insights into the significance of structural biology to containing the prevalent diseases in Africa. Specifically, the aim was to enable the participants to produce protein crystals by the traditional vapour diffusion method and remotely connect to a synchrotron beamline at the European Synchrotron Radiation Facility (ESRF) in Grenoble, France and collect X-ray diffraction data from crystals mounted on the beam by a beamline scientist. In addition, the participants were taken through the process of cryo-electron microscopy data collection and structural determination at the ESRF.

## Promoting structural biology in Africa

Africa is the only continent without a synchrotron light source (Connell et al., 2019; Wild, 2021), which has led to the establishment of the African Light Source Project to raise awareness and mobilise resources to construct the first African synchrotron light source (Connell et al., 2019). BioStruct-Africa's primary mission is to train, develop and maintain world-class structural biologists working in Africa on diseases predominantly affecting the African continent. BioStruct-Africa also supports the vision and roadmap towards building the first African synchrotron light source. The BioStruct-Africa initiative would ensure that by the time the continent's synchrotron light source is turned on, many Africans would have been trained to work on synchrotron science-related projects. The urgency of the BioStruct-Africa initiative is particularly relevant with the recent COVID-19 pandemic that led to the number of deaths from diseases like malaria rise due to the negligence and disruption of health services caused by the pandemic. In this light, BioStruct-Africa recently organised a workshop at MRTC. The goal of the workshop is to train Africa-based scientists to utilise structural biology in containing the diseases that are prevalent in Africa.

## Workshop framework

The BioStruct-Africa workshops are structured to contain both theoretical lectures and hands-on training in structural biology.

### Theoretical lectures

The workshop kick-started with an introduction to structural biology by Dr Michel Fodje (Canadian Light Source, Inc. Saskatoon, Canada). Dr Fodje's lecture mainly focused on the history of structural biology and the introduction of structural biology techniques, such as macromolecular crystallography, cryo-electron microscopy (cryo-EM), nuclear magnetic resonance spectroscopy, and small-angle X-ray scattering. Dr Fodje's lecture was followed by an overview of structural biology at the ESRF (Grenoble, France) by Dr Daouda A.K. Traore (Life Sciences Group, Institute Laue Langevin, Grenoble, France; slides provided by Dr Christoph Mueller-Dieckmann). The theoretical lecture session continued with two lectures back-to-back by Dr Emmanuel Nji [Centre for Research in Therapeutic Sciences (CREATES), Strathmore University, Nairobi, Kenya and BioStruct-Africa, Stockholm, Sweden]. In the first lecture, Dr Nji presented the strategies for selecting a suitable target for structural biology and the principles underlying a protein crystallization experiment. In the second lecture, Dr Nji presented the significance of structural biology for Africa. He specifically highlighted what approaches had been utilised that might prove useful in containing the prevalent diseases in Africa using a structure-based drug and vaccine design approach. Of particular interest during Dr Nji's second lecture was the number of structures solved in the pursuit of therapeutics for the COVID-19 pandemic in such a short time (more than 1000 COVID-19 viral protein structures were deposited in the protein data bank within one year from the start of the pandemic), highlighting the role played by structural biology in containing COVID-19 (Lubin et al., 2022; Lynch et al., 2021; Scudellari, 2020). Likewise, the importance of structural biology in understanding the molecular basis for drug resistance by the malaria parasite to chloroquine through the chloroquine resistance transporter (PfCRT) was of particular interest to the participants (Kim et al., 2019). Finally, the search for novel therapeutics to counteract malaria's growing drug resistance crisis by targeting the malaria parasite sugar transporter (PfHT1) was very revealing to the participants (Jiang et al., 2020; Qureshi et al., 2020). Dr Adeline Robin Traore from the European Molecular Biology Laboratory (Grenoble Outstation, France) gave a presentation on high-throughput crystallisation and structure-guided and fragment-based drug design. Next, Prof. Oluwatoyin A. Asojo (Chair of the Department of Chemistry and Biochemistry, Hampton University, Virginia, USA and scientific adviser of BioStruct-Africa, Stockholm Sweden) shared her experience with conducting structural science with undergraduates at Hampton University, a premier historically black college and university. Her participation in the workshop showcased to the young female participants that women of colour can be successful in structural biology. Her participation was particularly relevant, especially as we have had challenges getting the required number of female participants during our workshops. In addition, Dr Asojo's approach developed during the COVID-19 pandemic does not require infrastructures that are not available in the African settings. Her approach involves analysing, interpreting and preparing manuscripts for publication with structures solved and supplied by The Seattle Structural Genomics Center for Infectious Disease (https://www.ssgcid.org/). This workshop registered a higher number of female participants than the previous one, which could be because of our advertising strategy encouraging females to apply and providing a few stipends to support their participation.

Finally, Mikael Andersson Schönn (Bio-Works, Uppsala, Sweden) presented the tricks of the trade for protein purification for crystallography purposes. It is not typically expected of structural biology scientists to also be protein purification experts. As such, having a relevant source of expertise in the field might help save projects with issues that otherwise seem insurmountable. His participation and including the industry (Bio-Works, Uppsala, Sweden) in our course would help bridge the gap regarding the accessibility of consumables for protein purification experiments. Additionally, having a direct contact point to relevant companies through these workshops may provide future opportunities for university spin-off companies regarding industrial know-how.

### Hands-on training

**Fig. 1.**
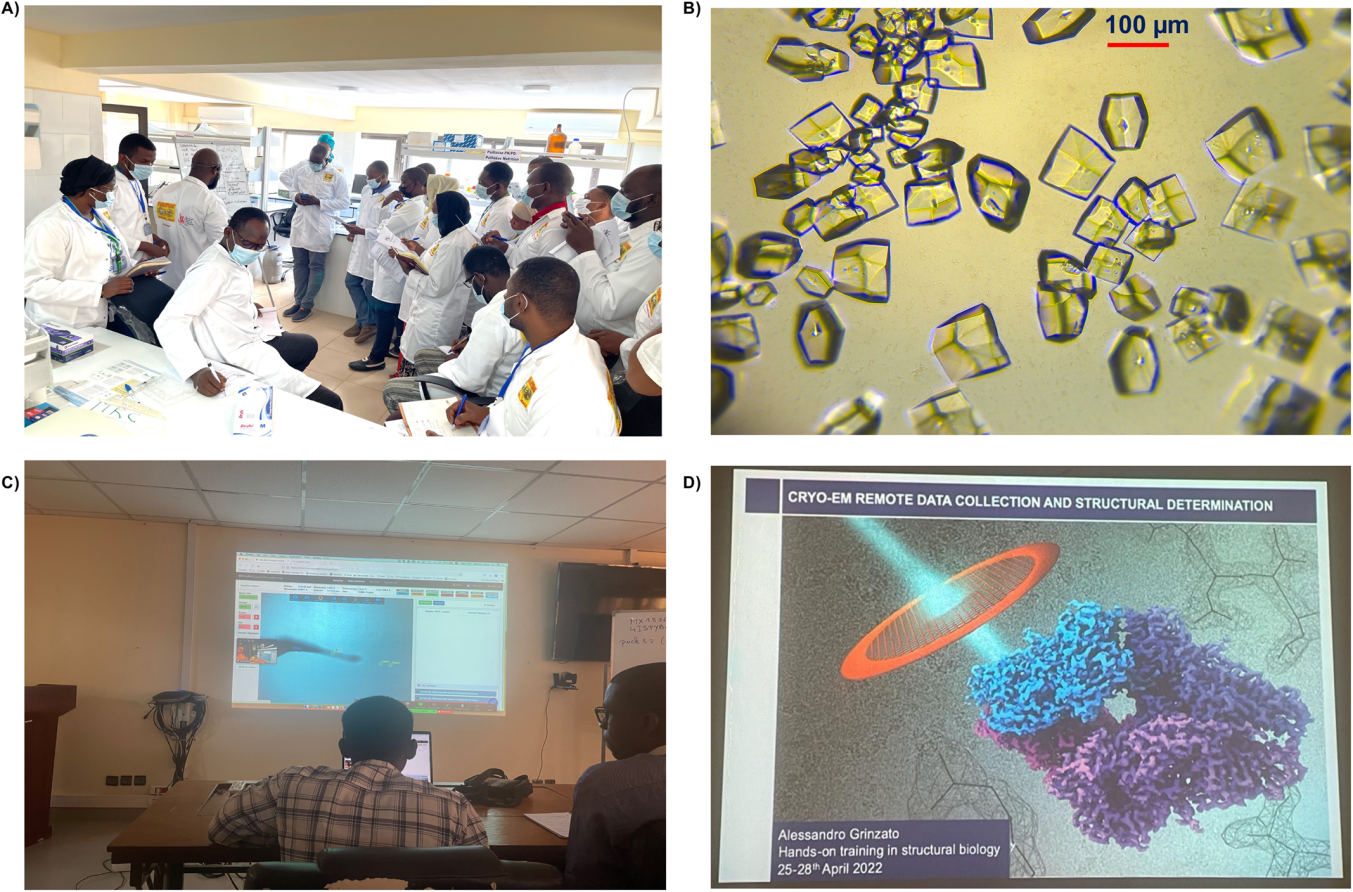
**Hands-on training in structural biology in Bamako, Mali from the 25th to 28th of April 2022.** (A) Introduction to the protein crystallization, crystal imaging, harvesting, and cryocooling lab session. (B) Crystals of lysozyme grown by hanging drop vapour diffusion. (C) X-ray crystallographic remote data collection. (D) Cryo-EM remote data collection and structural determination.

The participants were trained on vapour diffusion crystallization, crystal imaging, harvesting, and cryocooling using lysozyme as the protein of choice (Fig. 1A). After successful crystallization of the lysozyme protein solution (Fig. 1B), the participants were able to remotely connect to a beamline at ESRF and collect X-ray diffraction data from protein crystals mounted on the goniometer by a beamline scientist and determine the structure (Fig. 1C). Tutorials on crystallographic data analysis tools, such as Coot, protein data bank (PDB), and PyMoL, were also provided. Lastly, a tutorial on cryo-EM remote data collection and the subsequent structure determination process at ESRF was presented (Fig. 1D).

## Measuring impacts and achievements and recommendations from participants

**Fig. 2.**
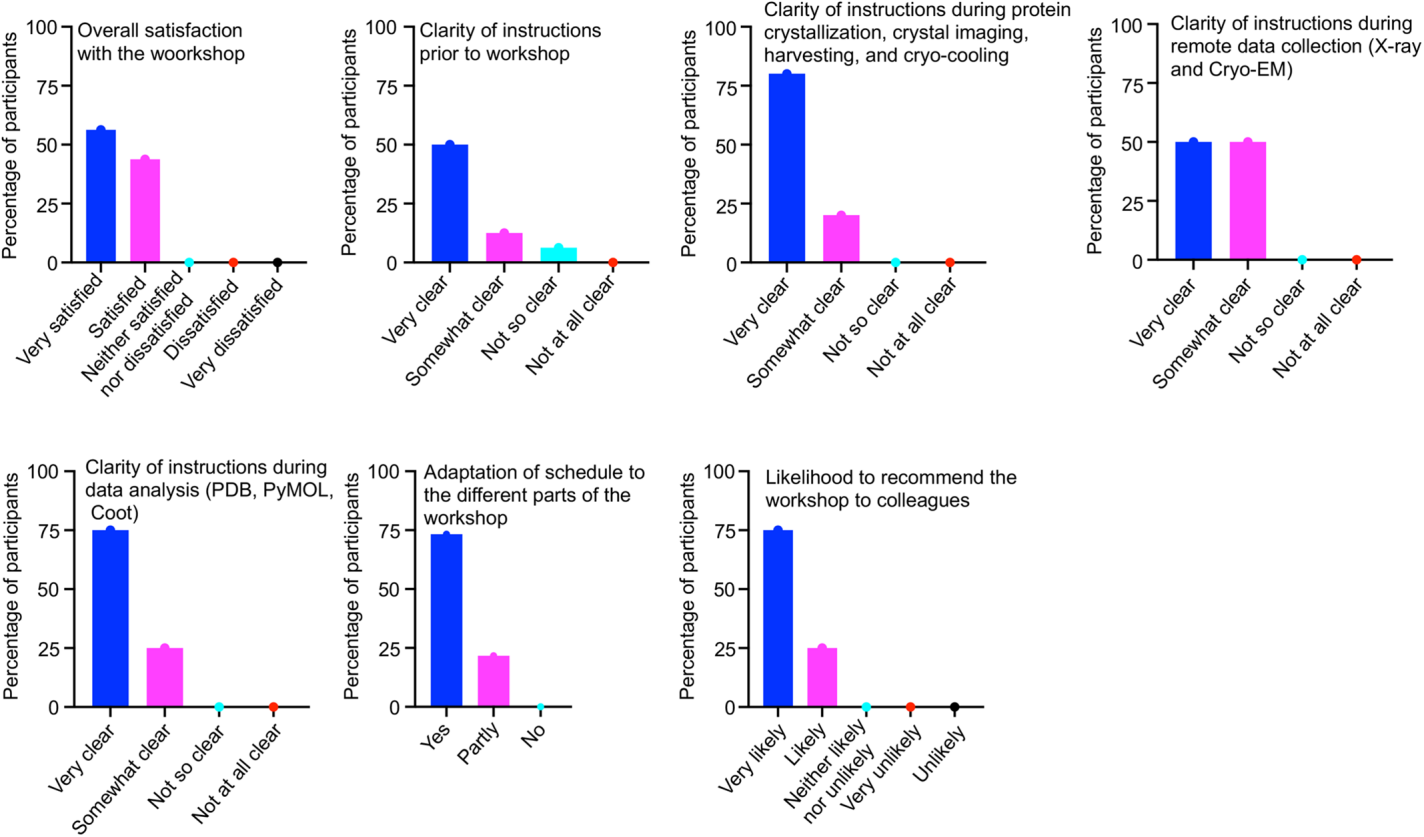
Workshop feedback report from sixteen participants.

The workshop was attended by 25 participants, mainly early-career scientists, Master’s and PhD students, mostly from Mali. The participants took a pre-test at the beginning of the workshop and, a post-test at the end to judge the success of our teaching strategy. Based on the pre- and post-test results and student presentations, it was evident that the participants benefited from the workshop. Likewise, participants were given an online survey to complete anonymously about the pros and cons of the workshop, and the results shown in Fig. 2. Overall, close to 60% of participants were very satisfied, and the remaining 40% were satisfied with the organization of the workshop. Likewise, 70% of the participants said they were very likely to recommend the workshop to their colleagues, while 30% were likely to recommend it. Most of the participants suggested we organise the workshop over a longer period. Specifically, some recommended the workshop be organised for five days rather than four.

## Concluding remarks

The vision of BioStruct-Africa is to ensure that the African continent actively conducts cutting-edge structural biology research. Research in structural biology would help provide the molecular details of disease intervention points and aid small molecule and vaccine development. Building capacity in structural biology for Africa-based researchers would ensure that Africans can utilise structural biology to find solutions to the diseases affecting their families, neighbours, and continent. In order to build capacity in structural biology for Africa-based researchers, BioStruct-Africa recently organised a workshop in structural biology at the MRTC, from the 25th to the 28th of April 2022. Overall, the workshop was very successful as participants were able to grow protein crystals and remotely connect to the European Synchrotron Radiation Facility, Grenoble, France, to collect X-ray diffraction data (Fig. 1). They also analyse and interpret a protein structure using tools such as Coot, PDB and PyMoL. The workshop feedback report (Fig. 2) showed that the participant benefitted from this inclusive and gender-balanced workshop. Most of the participants have already identified potential structural biology synergies in their research topics, which could open up avenues for future collaboration with the BioStruct-African team members and beyond. Such collaborations, in combination with establishing structural biology labs at African universities and institutes, would enormously facilitate the growth of structural biology in Africa. The availability of structural biology labs where the trainees can undergo further training towards a Master's or PhD degree and postdoctoral experience will be integral to ensuring sustainable capacity building. In future workshops, we plan to increase the duration to at least seven days, including about two to three days of hands-on training in protein expression and purification techniques. Also, we aim to include evaluation data on each presentation, presenter and instructor.
